# *Pseudomonas* diarrhea in a child suffering from acute lymphatic leukemia

**DOI:** 10.4103/0971-5851.65341

**Published:** 2009

**Authors:** A. De, H. Mathurkar, S. Baveja, M. V. Manglani

**Affiliations:** *Department of Microbiology, L.T.M. Medical College, Sion, Mumbai, India*; 1 *Department of Pediatrics, L.T.M. Medical College, Sion, Mumbai, India*

**Keywords:** *Acute lymphatic leukemia*, *Pseudomonas*, *diarrhea*

## Abstract

A female child admitted to hospital, diagnosed with acute lymphatic leukemia — CALLA positive, developed loose motions. Her stool culture and blood culture grew ***Pseudomonas aeruginosa***. Although the diarrhea subsided after five days, the stool culture repeatedly grew***P. aeruginosa*** for more than one month, in spite of treatment. Even though diarrhea due to ***Pseudomonas*** is rare, it can yet be seen in immunocompromised patients and is also associated with neutropenic enterocolitis. Stool specimens of all leukemia patients on chemotherapy and suffering from diarrhea, should be sent routinely for culture, so as to find out the exact cause of the diarrhea. Proper reporting will enable the clinicians to start appropriate antibiotics, thereby, reducing the morbidity and mortality of the leukemia patients.

## INTRODUCTION

*Pseudomonasis* notorious for causing hospital-associated infections, infection in burn patients, catheter-associated infections, chronic otitis media, eye infection, necrotizing pneumonia, septicemia in newborns and old debilitated persons, cystic fibrosis, malignancy, immunosuppression, and so on.[1] It rarely causes antibiotic-associated diarrhea,[2] however, it can cause diarrhea in immunocompromised patients[3] and is also associated with neutropenic enterocolitis in patients with acute leukemia, lymphoma, aplastic anemia, and so on.[4] We report here a case of *Pseudomonas diarrhea* in a child suffering from acute lymphatic leukemia.

## CASE REPORT

A one-year-three-month old female child, residing in Mumbai, was admitted in our hospital on 9 January, 2009, with complaints of high grade fever off and on, not associated with rigors, for the past one month. On examination, pallor was present along with ecchymosis and petechial rashes over the abdomen. She had hepatosplenomegaly. In the other systems, no abnormality was detected. Her hemoglobin was 5.7 gm%, TLC 16,400/cu.mm., with neutrophils 44%, lymphocytes 50%, and monocytes 6%. Platelet count was 8,000/cu.mm. Serum protein was 7 mg (albumin 4 and globulin 3), total bilirubin 1.1 mg/dl with direct bilirubin of 0.1 mg/dl. ALT 31, AST 13, and alkaline phosphatase was 337 IU/l, serum BUN 7 mg%, serum creatinine 0.7 mg%, serum sodium 138 mEq/l, potassium 4.1 mEq/l, calcium 8.9 mg%, and phosphate 4 mg%. She was diagnosed as a case of severe anemia with hepatosplenomegaly and acute lymphatic leukemia – CALLA positive. She was given chloroquine on admission and later two bottles of platelets were transfused. Antibiotics (Amikacin and Piperacillin-Tazobactam), Prednisolone (10 mg), Vincristine (0.36 mg), and Methotrexate (8 mg) were started. On 19 January, her hemoglobin increased to 7.7 gm%, TLC reduced to 7600/cu.mm, with neutrophils 27%, lymphocytes 68%, and monocytes 5%. The platelet count increased to 22,000/cu.mm. Two more bottles of platelet transfusion was given and the other medicines were continued.

On 27 January, she suddenly developed loose motions without abdominal pain. Stool was sent for culture along with blood culture in trypticase soy broth. After 24 hours, from the blood culture bottle, subcultures were peformed on MacConkey agar (MA) and blood agar (BA) plates and incubated at 37°C overnight. The stool was directly plated on MA and Xylose lysine deoxycholate agar (XLD). Pure non-lactose fermenting colonies with irregular margins grew on both the MA plates. They were oxidase positive and motile, and were identified as *Pseudomonas aeruginosa* by standard biochemical tests.[5] Thus the same organism was isolated from the blood and stool cultures. Antibiotic susceptibility was carried out using the Kirby-Bauer disk diffusion method on a Mueller Hinton agar, according to CLSI guidelines.[6] It was susceptible to amikacin, ofloxacin, piperacillin, and imipenem, and resistant to ceftazidime. The stool isolate, in addition, was susceptible to norfloxacin and resistant to nalidixic acid. On 27 January her complete blood count was by Hb 10.1 gm/dl, TLC 1100/cu.mm., P2, L8, Blasts90, Platelets 7,000/cu.mm.

She developed left facial palsy on 28 January and also developed perianal and gluteal abscesses.

On 29 January, she was put on amikacin, meropenem and metronidazole. Although the diarrhea subsided after five days, the stool cultures repeatedly grew *P.aeruginosa*. On 2 February, two stool samples were again sent for culture and both grew *Pseudomonas aeruginosa* in pure culture, with the same antibiotic susceptibility pattern, but the blood culture sent on the same day did not show any growth. On 12 February also, the stool culture grew *Pseudomonas aeruginosa*, susceptible only to amikacin. Blood cultures were further sent on 9, 16, and 20 February and 2 March. None of them showed any growth. Stool cultures were further sent on 20 February, 2 and 5 March, and they did not grow any pathogenic bacteria. She was given another bottle of platelet transfusion and was discharged on 9 March. At discharge, her condition was stable.

## DISCUSSION

*Pseudomonas aeruginosaas* a cause of infectious diarrhea is rare.[3] It usually represents a nosocomial infection in an immunocompromised host.[3] In PubMed Search with ‘*Pseudomonas* diarrhea in acute lymphatic leukemia’, no item was found. This child was already on chemotherapy. Mucosal damage is a major risk factor for complication of cytotoxic chemotherapy. Mucosal barrier injury in the gastrointestinal tract or gut mucosal ulcerations may result from direct drug-related cytotoxicity or from neutropenia itself.[7]

The normal fecal microflora changes in chemotherapy-induced diarrhea, in many patients, show a decreased proportion of anaerobic bacteria with a higher proportion of aerobic and oxygen-tolerant bacteria.[8] This child was already on chemotherapy when the diarrhea developed and she was already diagnosed to be suffering from acute lymphatic leukemia. It was probable that the normally reduced nature of the lumen became oxidized following chemotherapy, inhibiting anaerobic bacteria and allowing the growth of oxygen-tolerant bacteria.

More than 40% of *P.aeruginosa* had the ability to colonize in the human intestine and cytotoxic activity had been demonstrated in > 80% of *Pseudomonas aeruginosa* isolates.[8] Some of the isolates were weakly enterotoxigenic in infant mice and few also induced symptoms and signs of enteritis in clindamycin pre-treated rats.[9]

Neutropenic enterocolitis (NE) is a fulminant necrotizing process involving segments of the large and small intestine that occurs in the setting of agranulocytosis, commonly seen in patients with hematological malignancies, especially acute leukemia.[4] Agarwal *et al*. have reported *E.coli* and *Klebsiella* from the stool culture of a leukemic child, who developed NE.[4] The present case grew *Pseudomonas aeruginosa* from the stool as well as blood culture in a leukemic child. Although fever and diarrhea were present in this case, abdominal pain was absent, which was one of the hallmarks of NE. *Pseudomonas* was also reported to cause anorectal necrotizing ulceration in cases of acute leukemia.[10]

Therefore, stool specimens of all leukemic patients suffering from diarrhea, and on chemotherapy, should be sent routinely for culture, so as to find out the exact cause of diarrhea. Apart from stool culture, blood culture is also recommended in such cases. A high index of suspicion is needed for all patients who present with fever and abdominal pain in the setting of neutropenia.[7] Proper reporting will enable the clinicians to start appropriate antibiotics, thereby reducing the morbidity and mortality of leukemic patients, as was observed in this case.
